# Association between depression and metabolic dysfunction-associated steatotic liver disease: a cross-sectional analysis from the paracelsus 10,000 Study

**DOI:** 10.1007/s12072-025-10922-3

**Published:** 2025-10-01

**Authors:** Florian Koutny, Vanessa Frey, Christian Datz, Sophie Gensluckner, Julian Prosenz, Patrick Langthaler, Andreas Maieron, Maria Flamm, Daniel Weghuber, Bernhard Iglseder, Eugen Trinka, Bernhard Paulweber, Elmar Aigner, Bernhard Wernly

**Affiliations:** 1 Karl Landsteiner University of Health Sciences, Vienna, Austria; 2 Department of Internal Medicine 2 – Gastroenterology & Hepatology, University Hospital St. Pölten, St. Pölten, Austria; 3 Medical Science Research Program, Paracelsus Medical University, Salzburg, Austria; 4 Department of Neurology, Christian Doppler University Hospital, Paracelsus Medical University and Centre for Cognitive Neuroscience, Ignaz-Harrer-Straße 79, 5020 Salzburg, Austria; 5 Neuroscience Institute, Christian Doppler University Hospital, Paracelsus Medical University and Centre for Cognitive Neuroscience, Ignaz-Harrer-Straße 79, 5020 Salzburg, Austria; 6 Department of Internal Medicine, General Hospital Oberndorf, Teaching Hospital of Paracelsus Medical University, Paracelsusstraße 37, 5110 Salzburg, Austria; 7 University Department of Internal Medicine I – Gastroenterology and Hepatology, Nephrology, Metabolism and Diabetology, University Hospital Salzburg, Paracelsus Medical University, Strubergasse 21, 5020 Salzburg, Austria; 8 Institute of General Practice, Family Medicine and Preventive Medicine, Paracelsus Medical University, Strubergasse 21, 5020 Salzburg, Austria; 9 Center for Public, Health and Health Research, Paracelsus Medical University, Strubergasse 21, 5020 Salzburg, Austria; 10 Department of Geriatric Medicine, Christian Doppler Hospital, Paracelsus Medical University, Ignaz-Harrer-Straße 79, 5020 Salzburg, Austria; 11 Department of Public Health, Health Services Research and Health Technology Assessment, UMIT – Private University for Health Sciences, Medical Informatics and Technology, Eduard-Wallnöfer-Zentrum 1, 6060 Hall in Tirol, Austria

**Keywords:** MASLD, Depressive symptoms, Fatty liver index, Metabolic dysfunction, Steatotic liver disease, Liver fat accumulation, Population-based cohort, Mental health, Antidepressant use, Interaction analysis

## Abstract

**Background and aims:**

Depression is linked to obesity and metabolic syndrome, but its independent association with metabolic dysfunction-associated steatotic liver disease (MASLD) remains unclear. We examined the association between depressive symptoms and MASLD, independent of other risk factors.

**Methods:**

We analyzed cross-sectional data from 7433 participants of the Paracelsus 10,000 study. Depression (BDI-II > 13) and MASLD (FLI ≥ 60 plus ≥ 1 cardiometabolic risk factor) were assessed. Associations were examined using Poisson and linear regression models adjusted for demographics, lifestyle factors, metabolic syndrome, and antidepressant use. Subgroup and interaction analyses explored effect modification.

**Results:**

MASLD was more prevalent in participants with depressive symptoms than those without (37% vs. 27%, p < 0.01). Depressive symptoms were independently associated with MASLD (adjusted incidence rate ratio: 1.25, 95% CI: 1.13–1.39, p < 0.01), with consistent findings using continuous BDI-II scores. The association remained robust across subgroups defined by age, metabolic syndrome, education, smoking status, and antidepressant use. No significant differences were observed in fibrosis markers (APRI and FIB-4).

**Conclusion:**

In this large, population-based cross-sectional study, depressive symptoms were found to be associated with MASLD, independent of metabolic risk factors and antidepressant use. However, given the observational design and limited detail on antidepressant type, dose, indication, and adherence, the findings should be interpreted with caution and do not support causal conclusions. Nevertheless, findings suggest that mental health considerations may be relevant in MASLD screening and prevention strategies. Further research is needed to explore whether and how antidepressant therapy may relate to liver health.

**Graphical abstract:**

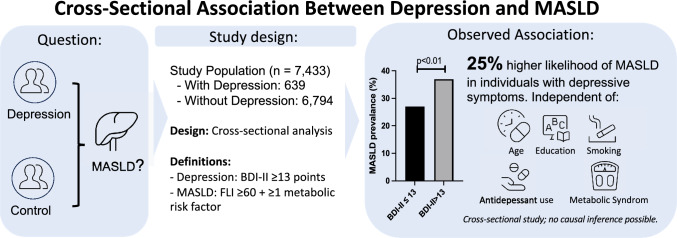

## Introduction

The rising prevalence of metabolic dysfunction-associated steatotic liver disease (MASLD) is becoming a growing challenge for health systems worldwide due to its association with cirrhosis, hepatocellular carcinoma, and increased healthcare costs [1]. MASLD is strongly linked to cardiometabolic risk factors such as type 2 diabetes, hypertension, and dyslipidaemia, which are commonly clustered under the umbrella of metabolic syndrome[1]. Recent research from our cohort has highlighted that the MASLD risk is influenced by socioeconomic factors, in particular educational attainment and income [2].

Depression has been increasingly recognized as a risk factor for morbidity and mortality across a wide range of chronic conditions, including obesity, hypertension, diabetes, coronary artery disease, and stroke [3–5]. Many of these comorbidities also co-occur with MASLD, suggesting a potential shared pathophysiology. In a large population-based study, Kim et al. reported a 1.6- to 2.2-fold increased odds of MASLD in individuals with depression [5]. Similarly, Cho et al. demonstrated that depression was associated not only with higher MASLD incidence, but also with advanced liver fibrosis, particularly in individuals with obesity [6].

Several biological pathways may underlie the link between depression and MASLD. Dysregulated stress responses have been associated with systemic inflammation, oxidative stress, and insulin resistance—all key contributors to hepatic steatosis [6–8]. Obesity may play a role in this relationship through elevated glucocorticoid levels, altered adipokine profiles, and pro-inflammatory signaling [8]. However, the link between psychological distress, metabolic dysfunction, and liver health remains incompletely understood.

In this population-based study, depressive symptoms were assessed using the Beck Depression Inventory-II (BDI-II), a widely used and robust self-report questionnaire that quantifies the severity of depressive symptoms across cognitive, affective, and somatic symptomes. MASLD was determined using the fatty liver index (FLI), a validated non-invasive score widely accepted for estimating hepatic steatosis in population studies In line with the new MASLD definition, the presence of at least one cardiometabolic risk factor—such as type 2 diabetes, hypertension, dyslipidemia, or central obesity—was also incorporated, to enhance diagnostic specificity. Liver fibrosis was assessed separately using the fibrosis-4 (FIB-4) index. [9–11] We aimed to investigate whether depressive symptoms are independently associated with MASLD, accounting for established demographic, metabolic, and lifestyle-related risk factors as well as antidepressant medication. We also explored whether this relationship applies to liver fibrosis. By exploring the link between mental health and MASLD, this study may help improve strategies to identify patients at risk and prevent disease progression.

## Methods

### Study design and population

This cross-sectional analysis utilized baseline data from the Paracelsus 10,000 Study, a population-based cohort study conducted between 2013 and 2020 [12]. This prospective registry was established to track the health status of individuals aged 40–77 years living in and around Salzburg, Austria. Participants were randomly selected from the local population registry, with equal representation of men and women, independent of any family structure [12]. The study protocol adhered to the Declaration of Helsinki and was approved by the local ethics committee (reference number 415-E/1521/6–2012). [12]

### Inclusion and exclusion criteria

**Fig. 1:**
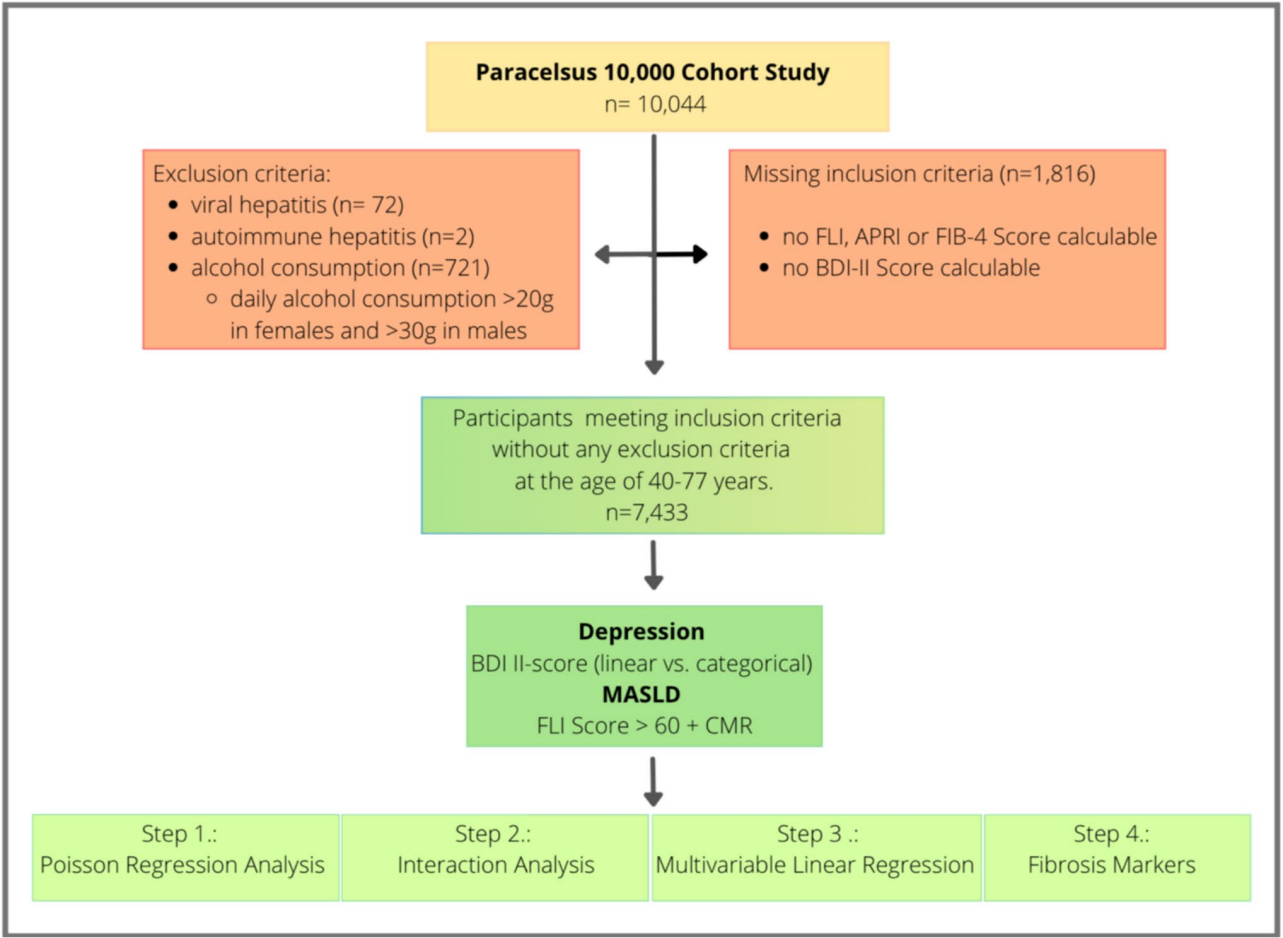
Flow chart illustrating the inclusion and exclusion criteria for the cross-sectional analysis of participants from the Paracelsus 10,000 cohort study. *FLI* fatty liver index, *APRI* AST to platelet ratio index; *FIB-4* fibrosis-4 score, *BDI-II* Beck depression inventory-II, *MASLD* metabolic dysfunction-associated steatotic liver disease, *CMR* cardiometabolic risk, *n* number of participants

Participants were included if they had complete data for fatty liver index, AST to platelet ratio index, and fibrosis-4 score calculations, as well as Beck Depression Inventory-II scores, daily alcohol consumption data, and information on antidepressant or tranquilizer use. To ensure that the study population exclusively reflected cases of MASLD and to rule out a primarily alcohol-related etiology, individuals with excessive alcohol intake—defined as more than 20 g per day for women or more than 30 g per day for men—were excluded. Additional exclusion criteria comprised the presence of viral hepatitis and missing data for any of the required variables. A detailed flow chart illustrating the participant selection process is provided in Fig. 1.

### MASLD definition and assessment tools

In this study, MASLD was diagnosed when two criteria were met: a fatty liver index (FLI) greater than 60 and the presence of at least one cardiometabolic risk factor, in line with the current MASLD definition and reflecting the metabolic basis of the disease. The fatty liver index is an important and practical tool because it is a well-validated, non-invasive score that enables reliable detection of hepatic steatosis, making it suitable for large-scale population studies and clinical screening. The score was calculated using the formula established by Bedogni et al., incorporating triglycerides, body mass index, gamma-glutamyl transferase, and waist circumference measurements. This validated score ranges from 0 to 100, with values of 60 or higher indicating hepatic steatosis with high sensitivity and specificity in population-based settings [11, 13].

According to the new MASLD definition, we additionally applied the requirement of a cardiometabolic risk factor in our definition of MASLD to further specify the diagnosis and enhance the precision of our findings. These cardiometabolic criteria included body mass index of 25 kg/m^2^ or greater, waist circumference of 94 cm or greater in men or 80 cm or greater in women, fasting glucose of 5.6 mmol/L or higher or existing type 2 diabetes or antidiabetic treatment, blood pressure of 130/85 mmHg or higher or antihypertensive treatment, plasma triglycerides of 1.70 mmol/L or higher, or plasma HDL-cholesterol below 1.0 mmol/L in men or below 1.3 mmol/L in women. [14]

Liver fibrosis was evaluated using two validated non-invasive markers. The AST to platelet ratio index was calculated as AST divided by the upper limit of normal, multiplied by 100, and divided by platelet count expressed as 10⁹ per liter. Values below 1.5 were considered to exclude significant fibrosis due to the high negative predictive value of this threshold [15, 16]. The fibrosis-4 index was derived using age, AST, ALT, and platelet count, with a threshold of 1.75 used to identify advanced fibrosis, corresponding to liver stiffness of 10 kPa or greater [17].

Depression was assessed using the Beck Depression Inventory-II, a widely validated 21-item questionnaire that measures the severity of depressive symptoms over the preceding two weeks. Scores range from 0 to 63, calculated as the sum of all items (0–3 each), with higher scores indicating greater symptom severity. The BDI-II was administered as part of the lifestyle assessment in the Paracelsus 10,000 study, requiring complete responses for inclusion. Clinically relevant depressive symptoms were defined as a BDI-II score > 13, consistent with prior validation studies in community samples. This threshold provides a good balance of sensitivity (81%) and specificity (92%) for detecting major depression and is widely recommended in psychometric reviews [9, 18].

### Clinical and laboratory assessments

Anthropometric measurements, including height, weight, and waist circumference, were obtained by qualified clinical personnel using standardized protocols routinely applied in a university hospital setting. Height was measured using a stadiometer, with participants standing without shoes. Weight was recorded using calibrated digital scales, with participants wearing light indoor clothing. Waist circumference was measured to the nearest 0.1 cm at the midpoint between the lowest rib and the iliac crest, with the participant standing and breathing out gently. All measuring devices were subject to regular quality control, including periodic inspection by the Technical Inspection Association (TÜV) and calibration performed according to the manufacturer’s specifications.

Body mass index was calculated as weight in kilograms divided by height in meters squared. Metabolic syndrome was diagnosed according to the revised National Cholesterol Education Program criteria when three or more of the following components were present: waist circumference of 102 cm or greater in men or 88 cm or greater in women, triglycerides of 150 mg/dL or higher, HDL-cholesterol below 40 mg/dL in men or below 50 mg/dL in women, blood pressure of 130/85 mmHg or higher or current antihypertensive treatment, and fasting glucose of 100 mg/dL or higher or current antidiabetic treatment [19].

Laboratory analyses were performed using standardized automated procedures and included comprehensive metabolic panels, liver function tests, and lipid profiles. Blood samples were collected after an overnight fast of at least 8 h. Diabetes was defined as haemoglobin A1c (HbA1c) levels exceeding 6.4% or current use of insulin or other glucose-lowering medications. Alcohol consumption was assessed using a standardized questionnaire in which participants reported their typical weekly intake, detailing the frequency and quantity of beer, wine, and spirits consumed. Antidepressant use was recorded as a binary yes/no variable without information on class, dose, duration, indication, or adherence.

### Statistical analysis

All statistical analyses were performed using Stata/IC 17 software. Statistical significance was set at p values less than 0.05 for all comparisons.

The primary objective was to investigate the association between depressive symptoms and MASLD using Poisson regression models to estimate incidence rate ratios with 95% confidence intervals. Three models were constructed to control for potential confounding factors. Model 1 provided unadjusted estimates, Model 2 included adjustments for age and sex, and Model 3 additionally controlled for metabolic syndrome, income level, educational attainment, smoking status, and antidepressant use. Covariate selection was based on a theoretical framework informed by clinical experience and supported by prior epidemiological and MASLD literature, rather than solely on statistical significance in bivariate analysis [2, 20]. The fully adjusted model includes age, sex, metabolic syndrome, income level, educational attainment, smoking status, and antidepressant use, as these variables are well-established risk factors for both depression and MASLD.

Secondary analyses examined depression both as a continuous variable using total Beck Depression Inventory-II scores and as a binary variable using the established cutoff of 13 points. Interaction analyses were conducted to test whether the association between depressive symptoms and MASLD varied across subgroups defined by sex, age, presence of metabolic syndrome, educational level, smoking status, and antidepressant use. Each interaction term was tested separately by adding it to the fully adjusted Poisson regression model. Sensitivity analyses were conducted by stratifying the association between depressive symptoms and MASLD by antidepressant us. Additional exploratory analyses examined potential mediation through metabolic syndrome by comparing models with and without metabolic syndrome as a covariate and performing stratified analyses by metabolic syndrome status. Linear regression models were used to assess the association between continuous Beck Depression Inventory-II scores and fatty liver index values as a continuous outcome, following the same stepwise adjustment strategy.

Due to their non-normal distribution, fibrosis markers including the AST to platelet ratio index and fibrosis-4 scores were analyzed as categorical variables using chi-squared tests to compare proportions between groups with and without depressive symptoms.

## Results

### Baseline characteristics

Of the 7433 participants included in this analysis, 6794 (91%) had BDI-II scores of 13 or lower, while 639 (9%) scored above 13, indicating elevated depressive symptoms suggestive of clinical relevance. The BDI-II scores showed the following distribution: mean = 4.96 (SD = 6.23), median = 3.0, range = 0–56. The distribution showed positive skewness (skewness = 2.27), which is consistent with expected patterns in general population samples where most individuals score in the lower range (25th percentile = 0, 75th percentile = 7) with fewer individuals showing higher symptom levels. Baseline characteristics stratified by depression status are presented in Table 1. Participants with depressive symptoms demonstrated several unfavourable metabolic profiles compared to those without depression. Glycaemic control differed significantly between groups, with median HbA1c levels of 5.5% in the depression group versus 5.4% in those without depression (p < 0.01).

**Table 1:** Clinical and metabolic differences by BDI-II score (≤ 13 vs. > 13)

VARIABLE	**TOTAL**	**BDI-II ≤ 13**	**BDI-II > 13**	p-VALUE*
	N = 7,433	N = 6,794	N = 639	
AGE [YEARS]	54 (49–60)	54 (49–61)	54 (49–59)	0.12
GENDER [% = FEMALE] (N)	52% (3,881)	51% (3,477)	63% (404)	< 0.01
ALT [U/L] (SD)	21 (16–29)	21 (16–29)	21 (16–29)	0.60
AST [U/L] (SD)	23 (19–27)	23 (19–27)	22 (19–26)	0.054
GGT [U/L] (SD)	22 (15–34)	22 (15–34)	21 (15–32)	0.24
ALP [U/L] (SD)	62 (52–74)	62 (52–74)	64 (54–77)	0.60
CHOLESTEROL [MG/DL] (SD)	208 (184–233)	208 (184–234)	210 (183–236)	0.60
TRIGLYCERIDES [MG/DL] (SD)	95 (70–133)	94 (69–131)	104 (77–150)	< 0.01
HDL CHOLESTEROL [MG/DL] (SD)	62 (51–75)	62 (51–75)	59 (48–73)	0.02
LDL CHOLESTEROL [MG/DL] (SD)	139 (115–164)	139 (115–164)	138 (115–164)	0.85
BMI [KG/M^2^] (SD)	25 (23–29)	25 (23–28)	27 (23–30)	< 0.01
ABDOMINAL CIRCUMFERENCE [CM] (SD)	92 (83–100)	92 (83–100)	94 (84–104)	< 0.01
HBA1C [%] (SD)	5.4 (5.2–5.6)	5.4 (5.2–5.6)	5.5 (5.3–5.7)	< 0.01
TYPE 2 DIABETES [%] (N)	3 (196)	3 (171)	4 (25)	< 0.01
METABOLIC SYNDROME [%] (N)	20 (1,501)	20 (1,324)	28 (177)	< 0.01
FLI ≥ 60 [%] (N)	28 (2,058)	27 (1,821)	37 (237)	< 0.01
APRI SCORE ≥ 0.5 [%] (N)	4 (239)	4 (221)	3 (18)	0.44
FIB-4 SCORE > 1.75 [%] (N)	9 (593)	9 (554)	7 (39)	0.07
ANTIDEPRESSANT THERAPY [%] (N)	3% (204)	2% (134)	11% (70)	< 0.01

Median (IQR) or % shown

*N* number, *ALT* alanine aminotransferase, *AST* aspartate aminotransferase, *GGT* gamma-glutamyl transferase, *ALP* alkaline phosphatase, *HDL* high-density lipoprotein, *LDL* low-density lipoprotein, *BMI* body mass index, *HbA1c* glycated haemoglobin, *FLI* fatty liver index, *APRI* AST to platelet ratio index, *FIB-4* Fibrosis-4 score, *SD* standard deviation

*p-values: comparison between BDI-II ≤ 13 and > 13.

Lipid abnormalities were more prevalent among participants with depression, who exhibited higher triglyceride concentrations (104 mg/dL vs. 94 mg/dL, p < 0.01) and lower HDL cholesterol levels (59 mg/dL vs. 62 mg/dL, p = 0.02). However, no significant differences were observed in LDL cholesterol or total cholesterol levels between groups. Anthropometric measures revealed higher BMI values in participants with depression (27 kg/m^2^ vs. 25 kg/m^2^, p < 0.01) and increased waist circumference (94 cm vs. 92 cm, p < 0.01). Consequently, metabolic syndrome was more prevalent in the group with depression (28% vs. 20%, p < 0.01), as was type 2 diabetes (4% vs. 3%, p < 0.01).

Liver enzymes showed minimal between-group differences. ALT and GGT levels were comparable across groups, while AST levels were slightly lower in participants with depression (22 vs. 23 U/L, p = 0.054). Alkaline phosphatase values were modestly elevated in the group with depression, though this difference was not statistically significant (64 vs. 62 U/L, p = 0.60). Within our analytical sample, missingness was minimal: outcome variable (0.00%), main predictor (0.00%), demographics (0.00%), metabolic syndrome (0.06%), education (1.49%), and antidepressant use (0.47%).

### MASLD prevalence

**Fig. 2:**
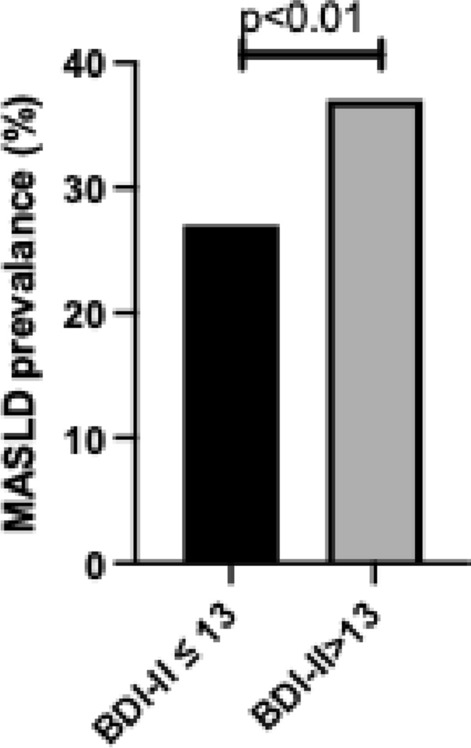
MASLD prevalence by depression status. Prevalence of metabolic dysfunction-associated steatotic liver disease (MASLD) among participants with Beck Depression Inventory-II (BDI-II) scores ≤ 13 (no depression) and > 13 (depression). MASLD was defined as fatty liver index ≥ 60 plus cardiometabolic risk factors. Data are presented as percentages. Statistical comparison was performed using chi-squared test. p < 0.01 indicates significant difference between groups

The prevalence of MASLD was significantly higher among participants with depressive symptoms compared to those without (37% vs. 27%, p < 0.01). Overall MASLD prevalence in the total cohort was 28%, as illustrated in Fig. 2.

### Primary association analysis

Table 2 presents the results of regression analyses examining the association between depressive symptoms and MASLD. When BDI-II scores were analyzed as a continuous variable, each one-point increase was associated with higher MASLD risk across all models. In the unadjusted analysis, the incidence rate ratio was 1.01 (95% CI: 1.01–1.02, p < 0.01). After adjustment for age and sex, the association remained significant. The fully adjusted model, controlling for metabolic syndrome, socioeconomic factors, lifestyle variables, and antidepressant use, yielded an IRR of 1.01 (95% CI: 1.00–1.01, p < 0.01).

**Table 2:** Association Between Depressive Symptoms and MASLD

*Model*	*IRR*	*95% CI*	*p-value*
**Continuous association**			
* Model 1*	1.01	[1.01, 1.02]	< 0.01
* Model 2*	1.01	[1.00, 1.01]	< 0.01
* Model 3*	1.01	[1.00, 1.01]	< 0.01
Categorical association (BDI-II > 13)			
* Model 1*	1.38	[1.24, 1.54]	< 0.01
* Model 2*	1.57	[1.42, 1.75]	< 0.01
* Model 3*	1.25	[1.13, 1.39]	< 0.01
Interaction analysis with BDI-II score (continuous)			
Interaction term	**IRR**	**95% CI**	**p-value**
Metabolic syndrome	1.00	[1.00, 1.02]	0.46
Age > 55	0.99	[0.98, 1.00]	0.20
Male	0.98	[0.97, 1.00]	0.02
Education (ISCED)			
Medium	0.99	[0.98, 1.00]	0.21
High	1.00	[0.98, 1.01]	0.69
Smoking status			
Previous smoking	1.00	[1.00, 1.01]	0.78
Current smoking	0.99	[0.98, 1.00]	0.15
Antidepressant use	1.00	[0.99, 1.01]	0.96
Linear regression			
Model	**β coefficient**	**95% CI**	**p-value**
Model 1	0.02	[0.01, 0.02]	< 0.01
Model 2	0.03	[0.03, 0.04]	< 0.01
Model 3	0.02	[0.01, 0.03]	< 0.01

Association between depressive symptoms (BDI-II) and MASLD: Poisson and linear regression models with BDI-II as continuous and categorical predictor. FLI used as continuous outcome in linear models. Models: 1: unadjusted; 2: + age, sex; 3: + metabolic syndrome, income, education, smoking, antidepressant use

*IRR* incidence rate ratio, *CI* confidence interval, *BDI-II* Beck Depression Inventory-II, *MASLD* metabolic dysfunction-associated steatotic liver disease, *ISCED* education level, *FLI* fatty liver index, *β* beta coefficient

**Fig. 3:**
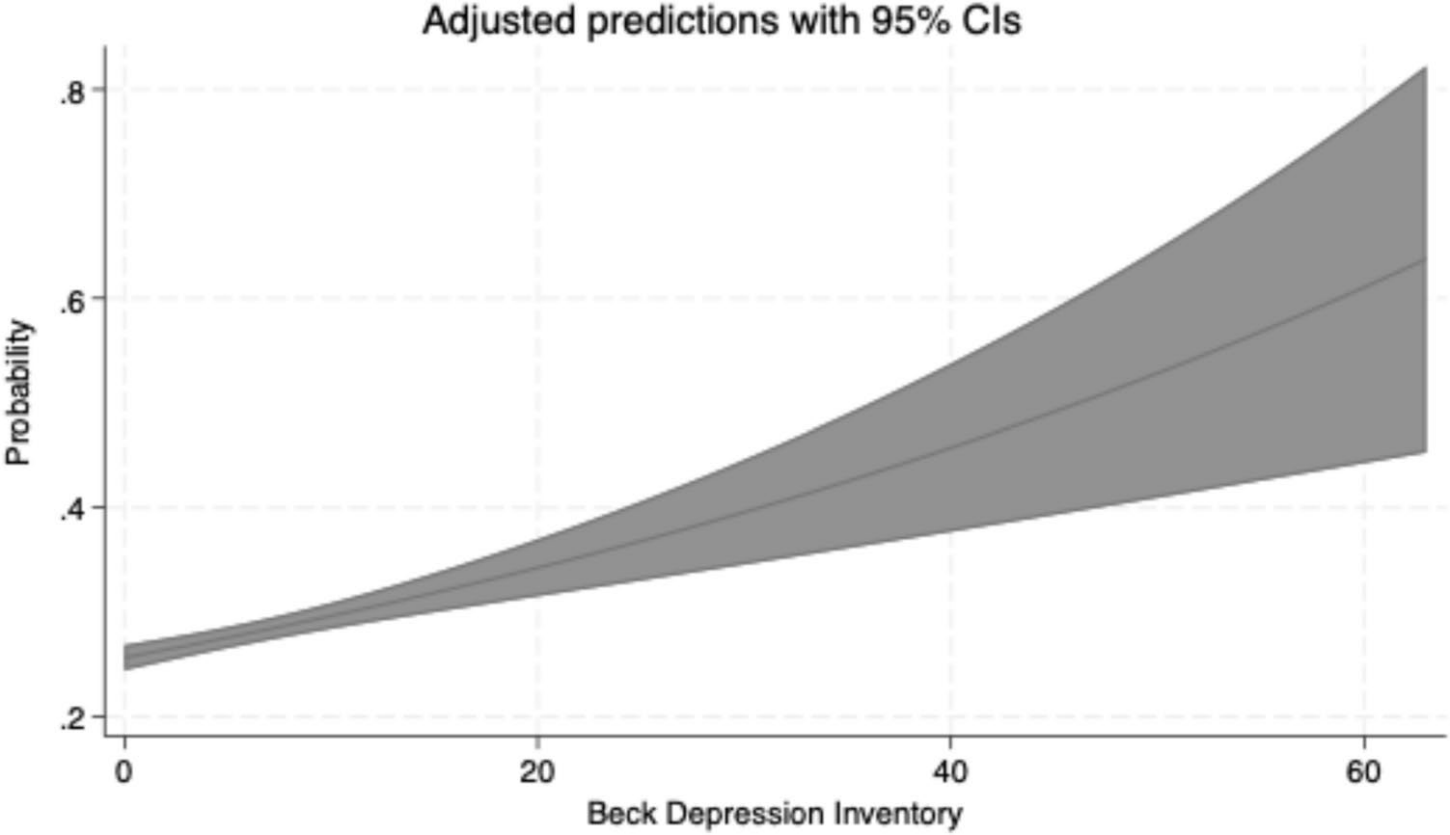
Adjusted probability of MASLD by BDI-II score. The graph shows predicted probabilities from a Poisson regression model adjusted for age, sex, education, smoking, metabolic syndrome, and antidepressant use. Shaded areas represent 95% confidence intervals

Binary analysis using the established BDI-II cutoff of 13 confirmed these findings. Participants with clinically relevant depressive symptoms showed a 38% higher MASLD risk in unadjusted analysis (IRR: 1.38, 95% CI: 1.24–1.54, p < 0.01). After age and sex adjustment, this association strengthened to an IRR of 1.57 (95% CI: 1.42–1.75, p < 0.01). In the fully adjusted model, the association remained robust with an IRR of 1.25 (95% CI: 1.13–1.39, p < 0.01), indicating a 25% increased likelihood of MASLD among participants with depressive symptoms. Figure 3 demonstrates the dose–response relationship between BDI-II scores and the predicted probability of MASLD.

### Interaction and sensitivity analyses

Interaction analyses revealed that the association between depressive symptoms and MASLD was generally consistent across subgroups. The relationship was not significantly modified by metabolic syndrome status (interaction IRR: 1.00, 95% CI: 1.00–1.02, p = 0.46), age over 55 years (IRR: 0.99, 95% CI: 0.98–1.00, p = 0.20), educational attainment, smoking status, or antidepressant use. Educational level showed no meaningful effect modification, with interaction IRRs of 0.99 (95% CI: 0.98–1.00, p = 0.21) for medium education and 1.00 (95% CI: 0.98–1.01, p = 0.69) for high education compared to low education. Similarly, smoking status did not significantly modify the association, with interaction terms of 1.00 (95% CI: 1.00–1.01, p = 0.78) for former smokers and 0.99 (95% CI: 0.98–1.00, p = 0.15) for current smokers. Antidepressant use showed no interaction effect (IRR: 1.00, 95% CI: 0.99–1.01, p = 0.96). However, a significant interaction was observed for sex, with the association being modestly attenuated in men compared to women (interaction IRR: 0.98, 95% CI: 0.97–1.00, p = 0.002).

In sensitivity analyses stratified by antidepressant use, the association between depressive symptoms and MASLD remained statistically significant both among participants without antidepressant therapy (IRR: 1.34, 95% CI: 1.18–1.53, p < 0.001) and among those with antidepressant therapy (IRR: 1.32, 95% CI: 1.05–1.67, p = 0.019). Results are shown in Table 3.

**Table 3:** Sensitivity analysis: BDI-MASLD association

*Model*	*No Antidepressant use (n* = *7,229)*			*Antidepressant use (n* = *204)*		
	**IRR**	**95% CI**	**p-value**	**IRR**	**95% CI**	**p-value**
Model 1	1.34	[1.18, 1.53]	< 0.01	1.32	[1.05, 1.67]	0.02
Model 2	1.54	[1.36, 1.75]	< 0.01	1.35	[1.07, 1.70]	0.01

*BDI-II* Beck depression inventory-II, *MASLD *metabolic dysfunction-associated steatotic liver disease, *IRR* incidence rate ratio, *CI* confidence interval

Stratified analyses by metabolic syndrome status revealed that the association remained significant in both participants without metabolic syndrome (IRR: 1.37, 95% CI: 1.12–1.68, p = 0.003) and those with metabolic syndrome (IRR: 1.09, 95% CI: 1.01–1.18, p = 0.026). The interaction analysis confirmed no significant effect modification by metabolic syndrome status (interaction p = 0.46), indicating that the strength of association between depressive symptoms and MASLD does not differ significantly between these groups.

### Secondary outcomes

Linear regression analyses using FLI as a continuous outcome variable supported the primary findings. In unadjusted analysis, each one-point increase in BDI-II score was associated with a β coefficient of 0.02 (95% CI: 0.01–0.02, p < 0.01). After age and sex adjustment, this association strengthened (β = 0.03, 95% CI: 0.03–0.04, p < 0.01), and remained significant in the fully adjusted model (β = 0.02, 95% CI: 0.01–0.03, p < 0.01).

### Fibrosis markers

Analysis of hepatic fibrosis markers revealed no significant differences between participants with and without depressive symptoms. APRI scores of 0.5 or higher were observed in 3% of participants with depression compared to 4% of those without depression (p = 0.44). Using a higher threshold of 0.7, elevated APRI scores were found in 7.3% of the depression group versus 6.1% of the non-depression group (p = 0.35). Similarly, FIB-4 scores showed no significant between-group differences. A FIB-4 score of 1.3 or higher was present in 8.4% of participants with depression compared to 7.9% of those without depression (p = 0.43).

## Discussion

This study identifies a consistent association between depressive symptoms and MASLD. Among participants with a BDI–II score > 13, the prevalence of MASLD, defined by an FLI score > 60 plus cardiometabolic risk, was significantly higher compared to those with lower depression scores (37% vs. 27%, p < 0.01). Comparable results were observed in a study by Prett et al. exploring the relationship between depression and obesity. They found that 43% of adults with depression were obese, compared with 33% of adults without depression [20]. Depression has been associated with subsequent weight gain and obesity, supporting the hypothesis that depressive symptoms may be relevant to the development of adverse metabolic outcomes over time [21, 22]. While our study is cross-sectional, these prior findings in related metabolic conditions strengthen the plausibility of the association we observed between depressive symptoms and MASLD.

Our findings were supported by regression models: as a continuous variable, each additional BDI-II point was associated with increased MASLD risk, and binary analysis confirmed a 25% higher risk in individuals with depressive symptoms in fully adjusted models. Interaction analyses showed no significant modification by age, metabolic syndrome, education, smoking status, or antidepressant use. The association was attenuated in men, yet remained statistically significant (interaction p = 0.002). Linear regression using FLI as a continuous outcome supported a dose–response relationship.

Importantly, the observed association between depressive symptoms and MASLD was independent of antidepressant use. To further examine whether this relationship varied by medication status, we first performed an interaction analysis, which revealed no significant effect modification by antidepressant use. This suggests that the association between depressive symptoms and MASLD does not differ substantially between individuals with and without antidepressant therapy. To explore this further, we conducted sensitivity analyses stratified by antidepressant status (Participants with and without antidepressant use). The association remained statistically significant in both groups, indicating that antidepressant use did not materially alter the observed relationship. However, due to the observational study design and the limited resolution of antidepressant data—which was recorded only as a binary yes/no variable without information on drug class, dose, duration, indication, or adherence—these findings are exploratory and do not allow conclusions regarding causal effects or pharmacologic mechanisms. In literature certain antidepressants, such as mirtazapine, have been associated with higher hepatic fat accumulation, while others may have neutral or even protective effects [23]. Given these heterogeneous profiles, future studies with detailed, class-specific and drug-specific medication data are needed to clarify the potential effects of antidepressants, different drug classes versus the impact of depression itself on liver health.

Regarding hepatic fibrosis, no significant differences were found between participants with and without depressive symptoms. The proportions of elevated APRI and FIB-4 scores were low in both groups. This limits interpretability, as low case numbers may reduce statistical precision. Kim et al. found depression to be a predictor of advanced fibrosis, but the effect diminished after adjusting for BMI, suggesting mediation through metabolic factors [24]. Given the low prevalence of advanced fibrosis in our cohort, our analysis cannot confirm or rule out an association of fibrosis with depression. However, results underscore the value of further studies in populations with a higher burden of fibrosis.

Biological mechanisms may explain the observed association. The accumulation of hepatic fat promotes systemic inflammation, insulin resistance, and oxidative stress. These pathways are also implicated in depression pathogenesis^,^ [7, 8]. Elevated free fatty acids, common in metabolic syndrome, activate CD4 + lymphocytes and upregulate chemokine receptor type 2 expression [8]. This facilitates neuroinflammation via increased blood–brain barrier permeability and microglial activation. On the other hand, impaired urea synthesis in MASLD may lead to ammonia accumulation, which, along with gut dysbiosis, may be involve in the gut–liver–brain axis and may predispose to neuropsychiatric manifestations [25]. These overlapping processes may lead to a two-way cycle between MASLD and depression.

Our study has several limitations. Du to the cross-sectional design, our findings represent associations (BDI-II and FLI) measured at the same time point and no conclusions can be drawn regarding the temporal or causal direction of the relationship between depressive symptoms and MASLD. Nevertheless, previous studies suggest that both directions are possible and longitudinal data are required to clarify causality [21, 22]. Furthermore, as the study population consisted of individuals living in and around Salzburg, Austria, the applicability of our findings to populations in different geographic, ethnic, or healthcare settings may be limited. The diagnosis of MASLD and fibrosis relied on non-invasive surrogate markers rather than imaging or histological confirmation, which may introduce misclassification. While we adjusted for alcohol consumption and smoking status to reduce confounding, other lifestyle factors or comorbidities, such as diet, physical activity, sleep quality, psychiatric comorbidities and medication adherence are more difficult to measure and were not available in our dataset. This may result in residual confounding, a common challenge in large-scale population studies. Additionally, self-reported lifestyle factors or information about alcohol consumption can lead to a reporting bias. Our data on other chronic liver diseases, including viral hepatitis or autoimmune liver disorders, were limited, which could contribute to further bias. Finally, we acknowledge that antidepressant use in our study was assessed as a binary yes/no variable, without information on drug class, dose, duration, adherence, or clinical indication. This limits interpretability and introduces a risk of confounding by indication, as antidepressants may have been prescribed for conditions other than depression. The association should therefore be interpreted with caution and does not allow conclusions about medication effects. Future studies with prescription registry linkage are required to differentiate medication effects from underlying psychiatric morbidity. However, the large, randomly sampled population enhances generalizability. The low prevalence of advanced fibrosis aligns with expectations in population-based samples but warrants cautious interpretation.

In summary, our findings indicate an association between depressive symptoms and MASLD in a large, population-based sample, independent of classical metabolic risk factors and antidepressant use. However, given the cross-sectional study design, no causal conclusions can be drawn. Antidepressant use was recorded only in binary form, without further clinical information (type, dosage, indication, etc.), making it impossible to determine whether the observed associations reflect underlying psychiatric morbidity, shared risk factors, or correlates with antidepressant use. Nevertheless, by highlighting a consistent link between mental health and liver health, our results underscore the importance of considering psychological factors in MASLD research.

## Data Availability

The datasets generated and/or analyzed during this study are available from the corresponding author upon reasonable request. The data are not publicly available due to licensing restrictions. Access may be granted with permission from LIFE Child, Leipzig, and the Leipzig Childhood Obesity Consortium.
